# Immune function and health outcomes in women with depression

**DOI:** 10.1186/1751-0759-4-3

**Published:** 2010-05-03

**Authors:** Cherie Howk, Mary Bennett

**Affiliations:** 1 Indiana State University, School of Nursing, 232 N Plum St, Clinton, IN 47842 USA; 2 Western Kentucky University, School of Nursing, 101 Lynn Rich Drive, Alvaton KY, 42122 USA

## Abstract

This research reports immune function and health outcomes in women with depression, as compared with a non-depressed control group. Using Psychoneuroimmunolgy theory and a descriptive comparison design, scores on the Beck Depression Inventory (BDI) were used to divide 40 non-hospitalized Caucasian women between the ages of 18 and 65 years into either the control or depression comparison group. Women with depression were found to report significantly more incidences of illness over the previous two months and they were found to have significantly more indicators of illness at the time of the exam as compared to the controls. However, contrary to what has been documented in some earlier studies of depression, women with depression were not found to have significantly different immune function measures as compared to the control group. There was also no significant correlation between scores on the BDI and natural killer cell cytotoxicity in this study. While these findings support a connection between depression and both increased self-report of illness and increased signs and symptoms of minor illness or inflammation on physical exam, this study was not able to document that these effects were related to decreased immune function, as measured by natural killer cell activity or white blood cell counts.

## Introduction

Depression is a biological, psychological, and social illness that affects roughly 15 million American adults in any given year. Depression costs billions of dollars in lost time, productivity, personnel replacement, medical care and, tragically, loss of life. The cost to women is disproportionally higher, with women representing about two-thirds of those affected [1]. In addition, at least one study demonstrated that women with depression have higher costs related to greater work absence than males with depression [2]. But is all of the morbidity from depression directly connected to the psychological aspects of the disease, or are people with depression actually more susceptible to physical illnesses?

According to Psychoneuroimmunolgy (PNI) theory, the central nervous system (CNS), peripheral nervous system (PNS), endocrine system, and immune system are part of an intricate communication and feedback system. Any action that causes change or illness in one part of this system, such as the CNS, can potentially cause changes in the other parts of the PNI system, such as endocrine or immune systems. Evidence suggesting that psychological stressors such as depression can alter immunological functions and possibly increase susceptibility to physical disease has accumulated over the past several decades [3].

### Studies of Depression and Immune Function

While there have been several studies indicating that depression may cause changes in immune function, studies documenting immune changes and actual health outcomes are rare. Therefore, the clinical significance of immune changes in depression is largely unknown. In addition, while most studies have found decreased immune function, particular in severely depressed hospitalized men, this finding has not been consistently supported in studies of non hospitalized women or those with less severe depression.

One early study of depression and immune function reported that persons with major depression actually may have increased numbers of NK cells in the blood, while other lymphocyte subset counts were not significantly different from that of control subjects [4]. Another small study found no differences in a number of immune system parameters using a mixed gender sample. There were no significant differences in cytokine measures such as IL-4, IL-10, TNF-alpha, IFN-gamma, no significance differences in levels of immunoglobulins, and no differences in the total number of NK cells, B cells, and T cells between persons with major depression (n = 37) and healthy control subjects (n = 15) [5]. A third early study found no significant difference in the number of lymphocytes or lymphocyte subsets between those with depression and those in the control group. There was also no difference in lymphocyte response to mitogens or NKCA. However, there were significant differences in subsets of the sample, with age, severity of depression, and hospitalization states being associated with significant immune changes. The authors concluded that changes in immune function may not be found in the overall population of those with depression, but might be seen in certain subsets, such as those who have more severe depression or are hospitalized [6].

As time and techniques improved, more studies started to include a wider range of outcome measures, including measurement of various inflammatory cytokines. In a small study of patients with major depression (n = 17), in addition to decreased NKCA, the patients also had increased IL-2 levels, compared with the control group (n = 10) [7]. IL-2 is a cytokine which increases inflammatory responses and normally increases NKCA, but even with higher levels of IL-2 the patients in this study still had lower natural killer cell functioning. Another study of 25 male patients with major depressive disorder documented both decreased NKCA and higher levels of circulating IL-6, another cytokine which increases inflammation and stimulates immune cell activity [8]. These outcomes indicate that the depressed subjects in these studies had both decreased cellular immune function (measured by NKCA) and increased inflammatory cytokines. This later finding could also be of clinical significance if it could be shown that these subjects had increased signs and symptoms of inflammation, but health outcomes were not documented in this study.

As can be inferred from the above studies, higher levels or counts of any given immune system component does not necessarily indicate better ability to resist disease or better health outcomes in general. According to a meta-analysis of several studies which measured both cell counts and the cell's ability to respond to disease causing organisms, it appears that while an increase in circulating white cells (usually neutrophils) is sometimes found in depression, these neutrophils demonstrate decreased phagocytic activity, indicating a decreased ability to defend the body against pathogens [9]. Therefore, assays which measure cellular cytotoxicity (such as measures of NKCA) may provide a better measure of the immune system's ability to provide an effective defense against disease.

Looking at studies of cellular immune function as measured by cytotoxicity or phagocytic response to mitogens, there is now accumulated data that documents generally decreased NKCA in persons with depression [8-12]. According to a meta-analysis by Irwin and Miller (2007), depression can lead to decreased cellular immune function as evidenced by decreased NKCA, decreased lymphocyte proliferation in response to mitogens, and decreased virus specific T cell responses. In addition, depression can cause increased inflammatory response from the immune system, as evidenced by increased overall WBC level and increased levels of pro-inflammatory cytokines [13]. Another important finding is that alterations in immune function seen in depression can be improved by successful antidepressant treatment [14-17]. For example, in a study of males with depression, successful treatment of the depression with an SSRI (citalopram) improved NKCA, while those who demonstrated no clinical response to the antidepressant treatment also had no improvement in NKCA [18].

Whether the above results hold true in female subjects is somewhat more debatable. Despite the relatively higher numbers of women with depression, early studies of immune function tended to avoid using female subjects, for fear that hormonal changes would interfere with the immune function results. A meta-analysis by Zorrilla et al. noted that studies of depression and immune function which included higher numbers of female subjects showed smaller decreases in NK cell functioning [9]. And a study by Motzer et al. (2002) of women with irritable bowel syndrome (IBS) and co-morbid depression reported no significant difference in NKCA between groups[19].

In summary, the literature to date demonstrates that most studies of depression and immune function report lower absolute NK cell counts and/or lower NKCA, particularly in the more seriously depressed and/or hospitalized populations [9]. However, depression related decreases in NKCA may be gender specific, as studies of men with depression have more consistently reported decreased NK activity, compared to studies of women with depression [9,20]. These findings and others provide the background and impetus for continued study of depression, immune function and health outcomes in women. The hypothesis tested in this study is that women with depression will have decreased immune function and increased symptoms of illness and/or inflammation, compared to women without depression.

## Methods

A descriptive comparative design was used to determine if there was a significant difference between depressed and non-depressed women on either immune function, as measured by NKCA and WBC with differentials, or health outcomes on a physical exam. Methods for this dissertation study were approved in 2000 by the Indiana State University Institutional Review Committee for the use of Human subjects. Community dwelling (non-hospitalized) women were solicited for inclusion in this study using a variety of methods, including community bulletin boards, churches, newspaper advertisements, e-mail, community mailing, and community clinics. The women were aware that the researcher was interested in the effects of depression on women's health, but that they did not have to be depressed to be included in the study. Participants were pre-screened by phone to determine if they met the inclusion and exclusionary criteria (see table 1). Those meeting the study criteria were scheduled for participation. The final sample was made up of 40 non-hospitalized women between the ages of 18 and 65 years old.

**Table 1:** Sample Inclusion and Exclusion Factors

**Inclusion Factors**	**Exclusion Factors**
Female	1. Minor surgery in the preceding two weeks
Age 18-65	2. Chronic illness
Non-Pregnant	3. Major surgery in the preceding six months
	4. Use of any street drugs or over 1 pack of cigarettes daily
	5. Use of alcohol over 10 ounces per week
	6. Taking immunosuppressant.
	7. Use of nutritional supplements other than one daily multivitamin and/or a calcium supplement
	8. Working the 11-7 shift in the past three days
	9. Exercising more than seven hours per week
	10. Fear of having blood drawn for lab testing

A comparison of demographic variables is displayed in table 2. The mean age of participants was 41 years old. Scores on the Beck Depression Inventory (BDI) were used to divide the participants into either the control or depression comparison group. Of the forty participants, 23 had BDI scores which indicated at least mild levels of depression, while 17 had scores which allowed them to be placed in the control group. The groups did not differ significantly by age, use of antidepressants, menstrual phase or menstrual day, BMI, number of office visits or use of antibiotics in the past two months.

**Table 2:** Comparison of Groups on Demographic Variables

	Mean for Depressed Group (n = 23)	Range for Depressed Group	Mean for Control Group (n = 17)	Range for Control Group	**Sig**.
Age	40.56 years old	18-63 years old	43.23 years old	18-65 years old	P = 0.524
BDI Score	24.34	11-51	3.52	0-9	P < = 0.001*
Menstrual Early Fol. Phase	2.13		2.35		P = 0.803
Menstrual Day	Day 7.47		Day 9.05		P = 0.632
Office visits in last 2 months	2 visits	0-30 visits	.411 visits	0-2 visits	P = 0.256
Number of Antibiotic used	.434 Rx	0-3 Rx	.117 Rx	0-1 Rx	P = 0.112
Missed Days work/school	0.652 days	0-3 days	0.058 days	0-1 days	P = 0.007*

### Data Collection and Procedures

After informed consent, participants completed the Beck Depression Inventory, the Health Questionnaire, had a health history and physical exam, urinalysis, CBC and blood drawn for the natural killer cell assay. The physical exams were performed by nurse practitioners who were blind to subject group, the natural killer cell assay was performed by a nurse researcher also blind to subject group. Persons who tested depressed on the BDI were offered assistance in accessing professional help if they so desired.

### Measures of immunity

Measurement of the total number of circulating leukocytes is a common and easy to conduct test and is frequently used in evaluation of various disease process. Therefore this test was included as a simple measure of immune function. A dipstick urine analysis was performed as another test for infection. Both urinalysis and WBC count were measured by routine laboratory guidelines. The blood was processed in an automated Coulter counter to determine the WBC count. A CLIA-CERTIFIED laboratory completed the standard WBC and differential.

In addition, a more specific measure of immune function, NCKA, was used to determine if there was significant difference in immune function between depressed and non-depressed women. For this study, a modified version of the NK assay was used, which involved K 562 target cells grown up in one culture, then frozen in lots for use during the entire study. One lot of the prepared target cells were thawed 72 hours before each assay, then split 24 hours before use in the assay. In our previous work a side by side comparison of this modified procedure with the standard procedure yielded a mean test-retest reliability of 0.904 for five control subjects over a five-week period, compared to a mean test-retest reliability of 0.777 for the standard 51 Cr release NK cell activity assay[21].

NK cell activity was measured using fresh donor cells in a modified version of the standard four hour chromium release assay. Triplicate data from the NK assay was examined to determine measures of central tendency and check for outliers. The data was accepted or rejected based on criteria developed by Stone, et al. [22]. The K562 cells were labeled and viability was ascertained before data collection procedures each day. If the K562 viability was less than 80% or the spontaneous release was greater than 10% of the maximum release, another batch of K562 cells would have been used. For this study the spontaneous release remained below 10% of the maximum release, and all targets had a viability greater than 80% on all days of the study. Natural killer cell cytotoxicity was determined for effector to target ratios of 40:1, 20:1, 10: and 5:1, which was then was converted into standardized lytic units (LU) using the method described in previous research [23].

### Measures of depression

Due to the ease of administration, the Zung self-rating depression scale was utilized for telephone prescreening of participants [24]. Scores on the BDI, which detects and assess intensity of depression, were used to assign participants into either the depression or control group. The BDI includes an assessment of 21 symptoms and attitudes that are rated from 0 to 3 in terms of intensity. It takes 5 to 10 minutes to complete and is scored by summing the ratings of each of the 21 items [25]. The total BDI score was utilized to determine the level of depression. Participants who scored from 0 to 9 were determined to be within the normal range and placed in the control group. Participants who scored between 10 and 18 were classified as mild to moderately depressed; 19 to 29, moderate to severely depressed; and 30 and above, extremely severely depressed. All participants that scored above ten were placed in the depressed group.

### Measures of health

The self-report Health and Lifestyle Questionnaire [26] was used to operationalize a wide variety of self reported health problems. Test-retest reliability coefficient for this instrument was reported as 0.89 in prior studies [27]. In addition to the self-report measure, a physical examination was performed by nurse practitioners to document signs and symptoms of current illnesses or infectious processes. Examiners were blinded to participant's group status. They followed an established protocol investigating for generalized infectious and/or inflammatory processes in the integumentary system, respiratory tract, gastrointestinal tract and genitourinary tract. A routine review of systems was also completed as part of the physical exam. Positives and negatives were recorded on the tool provided. Total positives were summed and used as the index score for physical exams. The higher the score the more signs and symptoms of illness, infectious and/or inflammatory processes were found.

### Statistical Methods

Pearson-r was used to examine relationships among the variables. A minimum sample size of thirty-two participants insured an 85% chance of detecting a population Pearson correlation coefficient of 0.5. Therefore, a sample size of 40 was adequate to provide valuable information in this initial investigation of depression, immune function and health. Student t-tests were utilized to compare the two groups on the different variables. However, it must be noted that the sample size afforded in this study was too small to obtain power for this analysis.

## Results

The hypothesis tested in this study was that women with depression would have decreased immune function and increased symptoms of illness and/or inflammation, compared to women without depression. The second part of this hypothesis was supported in this study. Student independent t-tests were used to document significant differences between the control and the depression group. The participants in the depression group were found to be significantly different than the controls on several of the health outcomes. Women in the depressed group had significantly more findings on the review of systems, physical exam, total physical exam, and number of days missed from school or work when compared with women in the comparison group.

Women with depression were found to report significantly more incidences of infection and illness over the previous two months than the controls (t = -5.05, p < = 0.001). Table 3 shows selected health outcomes for women in both groups. The mean numbers of illnesses experienced in the past two months by each group were: depressed group 188 and control group 53. Additional findings include significant differences between groups on the report of headache, upset stomach, nausea and vomiting, no energy and yeast infections. In each case the depressed group reported significantly more problems with these signs and symptoms in the past two months. In addition, at the time of the physical exam, women with depression were found to have significantly more positives on the review of systems (t = -4.01, p < = 0.001).

**Table 3:** Comparison of Incidence of Symptoms over Past Month.

Field	Mean Depressed (SD) (n = 23)	Sum Depressed	Mean Control (SD) (n = 17)	Sum Control	**Sig**.
Headache	24.48 (24.5)	563	9.94 (15.6)	169	P = 0.039*
Acne	4.91 (12.5)	113	.35 (.79)	6	P = 0.097
Upset Stomach	19.09 (23.7)	439	3.88 (5.27)	66	P = 0.007*
Ear Infection	.61 (1.7)	14	.06 (.24)	1	P = 0.151
Sinus Infection	4.83 (12.6)	111	.35 (.70)	6	P = 0.104
Sore Throat	2.00 (4.2)	46	.29 (.69)	5	P = 0.069
Allergies	22.09 (28.5)	508	9.41 (19.5)	160	P = 0.123
Tooth Abscess	.13 (.46)	3	0 (0)	0 (0)	P = 0.186
Nausea/Vomiting	6.65 (13.4)	153	.82 (1.98)	14	P = 0.054
Diarrhea	5.74 (12.5)	132	.76 (1.56)	13	P = 0.073
Muscle Strain	8.913 (17)	205	1.0 (2.12)	17	P = 0.038*
No Energy	39.17 (25.35)	901	6.65 (14.1)	113	P < = .001*
Skin Infections	4.78 (13.2)	110	.12 (.49)	2	P = 0.105
Yeast Infection	.22 (.52)	5	0 (0)	0	P = 0.057
Urinary Tract Infection	.26 (.45)	6	.06 (.24)	1	P = 0.076
Vaginal Infection	.09 (.29)	2	0 (0)	0	P = 0.162

Table 4 demonstrates the findings on the history part of the physical exam for women in both groups. Depressed women reported a mean of 8 positives while controls reported 3 positives. While the above findings could have been biased by being primarily self-reported data, women with depression were also found to have significantly more indicators of infection and illness at the time of the physical exam as compared to controls (t = -2.23, p < = 0.032).

**Table 4:** Comparison of groups on selected physical exam findings

Field	Mean Depressed (n = 23)	Sum Depressed	Mean Control (n = 17)	Sum Control	**Sig**.
Nutrition	.48	11	.53	9	P = .757
Inflammation	.09	2	.00	0	P = 0.102
Redness	.17	4	.00	0	P = 0.043*
Throat	.22	5	.41	7	P = .194
Sinus	.22	5	.18	3	P = .757
WBC in urine	.30	7	.06	1	P = 0.039*
Nitrites in urine	.09	2	.00	0	P = .162
RBC in urine	.13	3	.18	3	P = .696
Urine clarity	.09	2	.00	0	P = 0.162
Yeast	.22	5	.00	0	P = 0.022*

	**Mean Depressed**	**Range**	**Mean Control**	**Range**	**Sig**.

NCKA (Lytic Units)	29.4	1.9-84	17.01	Range 4.1-52.8	P = 0.082
WBC (cu mm)	6.56	3.3-9.9	6.14	4.2-7.9	P = 0.371
Symptom Days	188.04	28-378	53.94	0-231	P < 0.001*
Positives on PE	3.13	1-8	1.7	0-4	P = 0.032*
Positives ROS	8.39	1-16	3.58	0-9	P < 0.001*
Total PE Findings	11.52	4-23	5.41	0-13	P = <0.001*

Table 4 also demonstrates comparisons on selected physical exam findings and immune function outcomes for women in both groups. Nutritional deficit (above ideal body weight) was the indicator found most often during the physical exam for subjects in both the depressed (11) and non-depressed group (9). The next highest indicator found during the physical exam in the depressed group was WBCs found in the urine (7), following by yeast (5), sinus tenderness (5), and redness or exudate in the throat (5). In the non-depressed group, the second highest finding was redness or exudate in the throat (7), followed by sinus tenderness (3), redness or changes in the TM (3), and RBCs found in the urine (3). Results of the total physical exam (exam positives plus positives on the review of systems) revealed significant differences between the groups with depressed women having more overall positive indicators of infection and illness (t = -4.18, p < = 0.001). Controls had a mean of 5 positives while depressed women had 11 positives on exam.

However, the first part of the study hypothesis was not supported. Women with depression did not have significantly different natural killer cell activity (NKCA) when compared to the control group (t = -1.78, p = 0.082). Mean NKCA for those in the depression group was 29.43 lytic units, the control group had a mean NKCA of 17.01 lytic units. In addition, white blood counts were not significantly different between the groups (t = -.91, p = 0.371). In fact, no part of the WBC and differential counts were found to be significantly different between the groups.

Table 5 displays correlations examined in this study. Looking at all the subjects no significant relationships were found between scores on the BDI and NKCA (r = .194, p = 0.224), BDI and number of WBC (r = .077, p = 0.637), and BDI and number of objective findings on the physical exam (r = .194, p = 0.23). There were also no significant correlations between the immune function measures of NKCA or WBC and any of the other main variables. However, the total physical exam score (objective findings plus review of systems) was significantly correlated with the total self-report of illness (r = .582, p < = .001). There were also significant correlations found between scores on the BDI and total self-report of illnesses (r = .792, p < = 0.001), BDI and total physical exam score (objective findings plus review of systems) (r = .564, p < = 0.001), and BDI and positive findings on the review of systems (r = .615, p < = 0.001). Time depressed was also found to be significantly related to scores on the review of systems (r-.56, p = .00) and physical exam findings (r = .494, p = .001).

**Table 5:** Correlation matrix of variables.

Matrix	NKCA	WBC	Self Report	Beck	Time depressed	Review Symptom	Physical Exam	Exam Total	Age
NKCA		r = .097	r = .162	r = .196	r = .035	r = .119	r = .092	r = .124	r = .039
		p = 0.55	p = .319	p = .224	p = 0.83	p = .465	p = 0.57	p = .445	p = .811
									
WBC			r = .127	r = .077	r = .282	r = .257	r = .257	r = .187	r = -.24
			p = .436	p = .637	p = .078	p = .536	p = 0.11	p = .249	p = .134
									
Self Report				r = .792	r = .497	r = .597	r = .238	r = .582	r = -.17
				p = .00*	p = .001*	p = .00*	p = .076	p = .00*	p = .295
									
Beck					r = .608	r = .615	r = .194	r = .564	r = -.15
					p = .00*	p = .00*	p = .23	p = .00*	p = .354
									
Time depressed						r = .56	r = .494	r = .637	r = .068
						p = .00*	p = .001*	p = .00*	p = .676
									
Review Symptom							r = .354	r = .934	r = .01
							p = .025*	p = .00*	p = .951
									
Physical Exam								r = .66	r = .081
								p = .00*	p = .62
									
Exam Total									r = .022
									p = .893

## Discussion

Based on PNI theory and the prior work done in this field, it was hypothesized that women with depression would have decreased NCKA, and decreased health outcomes on physical exam, compared to a sample of non-depressed women. While there were some significant differences in health outcomes between the two groups, there were not significant differences in NCKA nor in WBC counts or differentials, compared to the women in the control sample.

### Depression and NCKA

The results of this study did not support that non-hospitalized women with mild to moderate depression have decreased NKCA. Earlier research completed primarily with hospitalized depressed males found significant depression of the NKCA, while subsequent studies of non-hospitalized female and mixed gender samples have not consistently reported decreased NKCA. Meta-analyses of depression and immune have noted that decreased immune function is more often reported in studies of the more severely depressed and/or hospitalized depressed subjects [9,13]. Therefore, inclusion of women who rated as "mild" depression on the BDI or the small sample size may also have contributed to the non-significant findings concerning NCKA in this study.

### Physical Exam Outcomes

As expected, women with depression were found to self-report more illness indicators. In addition, they had more positive physical findings on physical exam than nondepressed participants. The more depressed a person reported being, the more illness indicators they reported. The symptoms reported most frequently by the depressed group were; no energy, headache, allergies, upset stomach and cough. These are common symptoms seen by all primary care providers, in that the ten symptoms most commonly presented in primary care by persons with depression include: no energy, headache, anxiety, depressed mood, backache, insomnia, pains in the chest, dyspepsia, giddiness, pain in the trunk, arms and legs [28]. Such symptoms may originate from several sources. One explanation involves what has been called the "complainer syndrome" that is, the subjects who complain most about their health may also complain most about their mood.

In this research, consideration must be given to the notion that mental factors rather than organic factors may produce the symptoms reported. Psychological distress may increase care seeking for illness or infections independent of any effect of actual disease incidence. However, the positive findings on physical exam noted in this study were independent of these self-report factors. Subjects in the depressed group had more physical indications of illness and infection at the time of the exam. When noting the main physical findings of the depressed group, WBC's found in the urine, yeast infections and redness or exudate in the throat, it is difficult to attribute their physical exam finding to mental factors alone. In addition, the correlation data in this research found that the higher women scored on the BDI depression scale, the more health problems were reported and found on physical exam. Additionally, the longer a women was depressed the more physical findings were noted on exam.

Lastly, women with depression may have more physical illness simply because signs and symptoms of physical illness may have been overlooked by health care providers who were focused on treating the co-morbid diagnosis of depression. One cannot disregard the barriers that women with mental illness face when attempting to obtain sensitive and coordinated health care. It has long been recognized that primary care providers may routinely fail to diagnose illness and trauma in women with serious mental illness [29-31]. This often leads to inappropriate or inadequate treatment and increased costs. In one early study, general medical practitioners missed 32% of the physical problems and psychiatrists missed 48%. This same study also found that women had significantly more undetected medical diagnoses than men [29]. In a more recent study it was noted that that persons with mental illness continue to have high levels of undiagnosed or sub-optimally treated co-morbid physical illnesses [32]. The current changes in the health care delivery and financing systems present special problems for these women because the emphasis on cost containment can lead to brief interventions that may constitute less than adequate care.

## Conclusion

This research suggests that depression may play a role in the subsequent development of minor acute infectious and inflammatory conditions, as measured by outcomes on a brief physical exam. The mechanism by which depression influences theses outcomes remains unknown. This research found that women with depression had more self-report of illness and more physical evidence of illness and/or inflammatory response on exam as compared to controls. But the hypothesized pathway to illness, via alterations of natural killer cell function or white blood cell function, was not supported in this particular study. This study does not rule out the effect of depression on other immune system parameters, or the effect of depression on health outcomes via behavioral choices. Future research should include a wider variety of both behavioral measures and immune function outcomes to further delineate the effects of all of these factors on health outcomes in women with depression.

## Competing interests

The authors declare that they have no competing interests.

## Authors' contributions

CH recruited subjects, administered the psychological testing and performed physical exams, along with other health care providers. MB carried out the immunoassays, participated in the design of the study and performed the statistical analysis. CH drafted the manuscript. MB edited the manuscript for submission. Both authors read and approved the final manuscript.
